# Development of software for the calculation of kinetic parameters from solid phase reaction data, application to synthesized *N*’-(4-Chlorobenzoyl)-*N*,*N*-di-*N*-butylthiourea ligand and its nickel (II) complex

**DOI:** 10.55730/1300-0527.3438

**Published:** 2022-05-11

**Authors:** Esra KALEM, Nevzat KÜLCÜ

**Affiliations:** 1 Faculty of Arts and Sciences, Ondokuz Mayıs University, Samsun, Turkey; 2 Faculty of Arts and Sciences, Mersin University, Mersin, Turkey

**Keywords:** Thermodynamic parameters, software program, thermal analysis methods, thiourea, transition metal complexes

## Abstract

In gas and liquid phase reactions, the conversion of starting materials to products is directly controlled by the rate of the reaction, whereas in solid state reactions, it is determined by the rate of diffusion. When working with thermal analysis methods, tens of thousands of data to be obtained from measured differential thermal analysis (DTA), thermal gravimetry (TG), and differential thermal gravimetry (DTG) curves must first be digitized, and then these digitized data must be converted to concentrations and thermal values using appropriate software programs. Companies that produce devices using thermal analysis techniques generally adapt the encrypted software programs they use according to the needs of the user companies. Since the devices work like a black box, it is impossible for users to make any changes to the packaged programs. In addition, since the program cannot be seen, its logic cannot be understood most of the time. The program developed in this study will be put at the service of every researcher, and the system of the program can be used on any Windows Software compatible computer. While preparing the program, an easy-to-understand and flexible MATLAB environment was used. The thermal analysis data of synthesized *N*’-(4-chlorobenzoyl)-*N,N*-di-n-butylthiourea (HL) and its Ni(II) complex have been digitalized by measuring with thermal analyses such as DTA, TG with hardware technical equipment. By entering the raw experimental data taken into the prepared software program, thermodynamic data such as enthalpy and entropy can be calculated as well as kinetic parameters such as activation energy, reaction order, and rate constant. In this study, a software program was developed that can be used in the calculation of the desired thermodynamic parameters by using the experimentally measured ΔT, Δm, and dΔm values. The accuracy of the results was compared with the thermal results of the NaN_3_ reference material.

## 1. Introduction

Events such as sintering, melting, sublimation, or phase change can occur due to the increase in molecular, atomic, or ion movements when a pure solid substance is heated in an inert gas atmosphere [1, 2]. If intramolecular forces are weaker than intermolecular forces, matter can decompose to form new compounds [3, 4].

If there is more than one solid in the environment, there may be more interaction. As a result of double decomposition and addition reactions, new compounds can be formed, as well as solid solutions and eutectic mixtures [5, 6].

Thermal analysis methods such as TG and DTG are being used more and more each day to examine the kinetics of pyrolysis reactions [7]. Two basic kinetic methods are used in kinetic analysis. These are differential methods and integral methods.

In this study, the software program prepared based on the Broido Method is presented. By entering the raw experimental data received, besides thermodynamic data such as enthalpy and entropy, kinetic parameters such as activation energy, reaction order, and rate constant can also be calculated. In this study, a software program that can be used in the calculation of the desired thermodynamic parameters was developed by using the experimentally measured ΔT, Δm, and dΔm values.

MATLAB 5.2 environment was preferred in the study. MATLAB is a technical program developed for high-performance numerical computations and graphical programming. It offers numerical analysis, matrix operations, signal processing, and graphics drawing as a whole in an easy-to-use environment. MATLAB is derived from the words ‘MATrix LABoratuary’. In MATLAB, the basic element is a ‘matrix’ that requires no sizing. It is possible to solve many numerical problems in a very short time with MATLAB. It is also possible to write algorithms as in the Basic, Fortran, or C programming languages. MATLAB also includes a set of ready-made solution packages and functions written for specific applications, called ‘toolboxes’. Toolboxes consist of M-files needed to solve a problem. The algorithms used in MATLAB are based on the “least squares method” used in numerical integral and derivative calculations with the help of computers. It is possible to use MATLAB on PC, Macintosh, SUN, UNIX, VAX, Apollo, HP, DECStation, SGI, RS/6000, and Convex computer systems.

The prepared program was applied for calculating kinetic parameters of thermal decomposition of newly synthesized thiourea derivative and its complex. N’-(4-chlorobenzoyl)-N,N-di-n-butylthiourea and the Ni(II) complex of these ligands were synthesized and characterized. Decomposition knetic studies were carried out on the program prepared using thermal analysis data [8].

## 2. Calculation of thermal data of the ligand and complex

The ligand and its metal complex were synthesized and characterized as given in the literature [9]. HL and its nickel complex were studied by thermogravimetric analysis from room temperature to 1300 K in nitrogen atmosphere. DTA/TG/DTG diagrams of HL and NiL_2_ complex are shown in Figures 1a and 1b, respectively. From the TG curve of HL, it appeared that the sample decomposes in two stages in the temperature range of 403 to 802 K. The first decomposition occurs between 403 and 459 K, with a mass loss of 10.4%; the second decomposition starts at 459 K, and ends at 802 K, with an 88.9% mass loss. From the corresponding DTA profile, three endothermic peaks are noted, the first between 355 and 390 K, with a maximum at 362 K; the second between 390 and 443 K, with a maximum at 425 K; and the third between 443 and 600 K, with a maximum at 508 K. The first endothermic effect is related to the melting of HL (362 K). The other effects are due to the decomposition of the related compound. The TG curve of the NiL_2_ complex shows an initial mass loss in the temperature range of 501–612 K, corresponding to the decomposition of the complex to Ni(SCN)_2_. The mass loss at this stage is attributed to the evolved moieties di-n-butylbenzamide (theoretical mass loss: 75.4%, experimental mass loss: 75.7%). These agree with the literature data [9]. The last decomposition step occurs in the temperature range of 612–1273 K and it corresponds to the formation of Ni_3_S_2_ (theoretical mass loss: 88.7%, experimental mass loss: 88.4%) [9–16].

### 2.1. PC program for thermal decomposition kinetics

#### 2.1.1. Content of the program

Based on the Broido kinetic method in the program software, the graph of 1/T versus lnln(1/y) was drawn and a linear graph was obtained [17]. The desired kinetic parameters can be calculated from the obtained graph. The part up to the drawing of this graph is explained step by step.

Pure solids decompose when heated under vacuum, and at least some of the resulting decomposition products are volatile. The reaction can be monitored by continuously measuring the sample mass. Mass changes are evaluated with the following equality:


(2.21) $$\text{y}=\text{N}/{\text{N}}_{0}=({\text{W}}_{\text{t}}-{\text{W}}_{\text{s}})/({\text{W}}_{0}-{\text{W}}_{\text{s}})$$

In this equation, W_0_ is the initial mass, W_t_ is the mass at any time t, W_s_ is the final mass, and y is the mass fraction of the remaining material. If the pyrolysis is carried out in an isothermal environment, the rate of reaction is given by the following relation:


(2.22) $${\text{d}}_{\text{y}}/{\text{d}}_{\text{t}}=-{\text{ky}}^{\text{n}}$$

In this equation, n is the order of the reaction and k is the rate constant of the reaction. Rate constant according to Arhenius is given by the following equation:


(2.23) $$\text{k}={\text{Ae}}^{-\text{E}/\text{RT}}$$

In thermal analysis studies, temperature (T) is a linear function of time.


(2.24) $$\begin{array}{l} \text{T}={\text{T}}_{0}+\text{ut}\\ \text{dT}=\text{udt }\;\;\text{dt}=\text{dT}/u \end{array}$$

Here u [ΔT/Δt] gives the heating rate.


$$\begin{array}{l} {\text{d}}_{\text{y}}/{\text{d}}_{\text{t}}=-{\text{ky}}^{\text{n}}\;\;\;\text{or }\;\;\text{dy}/{\text{y}}^{\text{n}}=-(\text{A.}{\text{e}}^{-\text{E}/\text{RT}})\;\text{dt}\\ {\text{d}}_{\text{y}}/{\text{y}}^{\text{n}}=-\text{k dt }\;\;\text{or }\;\;\text{dy}/{\text{y}}^{\text{n}}=-(\text{A}/\text{u})\;{\text{e}}^{-\text{E}/\text{RT}}\;\text{dT}\\ \text{k}={\text{Ae}}^{-\text{E}/\text{RT}} \end{array}$$

If Equations (2.21–2.23) are combined, Equation (2.25) is obtained;


(2.25) $$\text{dy}/{\text{y}}^{\text{n}}=-(\text{A}/\text{u})\;{\text{e}}^{-\text{E}/\text{RT}}\;\text{dT}$$

If the integration of both sides of the obtained Equation (2.25) is taken;


(2.26) $${\;}_{\text{y}}\int_{\;}^{1}\text{dy}/{\text{y}}^{\text{n}}=-(\text{A}/\text{u}){\;}_{\text{y}}\int_{\;}^{1}{\text{e}}^{-\text{E}/\text{RT}}\;\text{dT}$$

has the expression. Pyrolysis reactions are generally first order. Equation (2.26) can be regulated as follows:


(2.27) $${\;}_{\text{y}}\int_{\;}^{1}\text{dy}/{\text{y}}^{\text{n}}={\;}_{\text{y}}\int_{\;}^{1}\text{dy}/\text{y}=-\text{ln y}=\text{ln}(1/\text{y})$$

The integration of the right-hand side of the equation was explored by Vallet and published as a monograph in 1961 [25]. According to this, the following :


(2.28) $$\text{If z}=\text{E}/\text{RT},\;\;\;\text{dz}=-\text{E}/\text{R}(1/{\text{T}}^{2})\text{dT}$$

Equation dT is written from Equation (2.28), and if the value of T is included in this Equation, Equation (2.29) is obtained:


(2.29) $$\begin{array}{l} \text{T}=\text{Rz}/\text{E }\;\;\text{dT}=-({\text{RT}}^{2}/\text{E})\text{dz}\\ \text{dT}=-({\text{R}}^{3}/{\text{E}}^{3}){\text{z}}^{-2}\text{dz} \end{array}$$

If the right-hand side of (2.26) is combined with (2.29), we get (2.30).


(2.30) $$\text{ln}(1/\text{y})=[-(\text{A}/\text{u})({\text{R}}^{3}/{\text{E}}^{3})]\;\int {\text{e}}^{-\text{z}}{\text{z}}^{2}\text{dz}$$

Solution of the integral part of Equation (2.30) is given,


$$\int {\text{x}}^{2}{\text{e}}^{\text{ax}}\text{dx}=(1/{\text{a}}^{3})({\text{a}}^{2}{\text{x}}^{2}-2\text{ax}+2)\;{\text{e}}^{\text{ax}}$$

If this expression is substituted at (2.30),


(2.31) $$\text{ln}(1/\text{y})=[-(\text{A}/\text{u})({\text{R}}^{3}/{\text{E}}^{3})]\;(-{\text{e}}^{\text{z}}({\text{z}}^{2}+2\text{z}+2))$$

is given by the expression. Integrate both sides of (2.31):


(2.32) $$\text{ln ln}(1/\text{y})=\text{ln }[-(\text{A}/\text{u})({\text{R}}^{3}/{\text{E}}^{3})]+\text{ln }(-{\text{e}}^{\text{z}}({\text{z}}^{2}+2\text{z}+2))$$

(2.33) $$\text{ln ln}(1/\text{y})=\text{ln }[-(\text{A}/\text{u})({\text{R}}^{3}/{\text{E}}^{3})]-\text{ln }{\text{e}}^{-\text{z}}+\text{ln}\;({\text{z}}^{2}+2\text{z}+2)$$

Substituting the value of z in Eq. (2.33) gives (2.34)


(2.34) $$\text{ln ln}(1/\text{y})=\text{ln }[-(\text{A}/\text{u})({\text{R}}^{3}/{\text{E}}^{3})]-\text{E}/\text{RT}+\text{ln}\;[{(\text{E}/\text{RT)}}^{2}+2\;\text{(E}/\text{RT)}+2]$$

Here


$$\begin{array}{l} \text{ln ln}(1/\text{y})=\text{m}-\text{E}/\text{RT}+\text{J}\\ \text{ln }[-{\text{(A}/\text{u)(R}}^{3}{/\text{E}}^{3}\text{)}]=\text{m}\\ \text{ln }{[\text{(E}/\text{RT)}}^{2}+\text{2 (E}/\text{RT)}+\text{2}]=\text{J} \end{array}$$

the expressions can be represented by m and J. When Equation (2.34) is examined, it is seen that the expression symbolized by j can be neglected next to the expression m. Accordingly, the final form of (2.34) can be given as (2.35):


(2.35) $$\text{lnln}(1/\text{y})=\text{ln}[-(\text{A}/\text{u})({\text{R}}^{3}/{\text{E}}^{3})]-\text{E}/\text{RT}$$

In Equation (2.35), the value of ln [−(A/u)(R^3^/E^3^)] is constant. Accordingly, the equation can be written as (2.36):


(2.36) ln ln(1/y)=-E/RT+constant

Plotting 1/T against lnln(1/y) results in a linear graph. (The linear plot of ln1/T versus lnln(1/y) gives good results in the range of 0.999 > y > 0.001.)

#### 2.1.2. Flow chart of PC program

The PC program was written and compiled using C++ programming language in the MATLAB 5.2 environment. Preparation of the program was in easy and flexible MATLAB concept. By using the TG data of the synthesized ligands and chelates, the program can perform the kinetic analysis of the pyrolysis reactions with the help of the Broido kinetic method [17].

In the program, only the percent amount of mass change and the relevant temperature from the TG analysis results are used as data. By using the prepared software program, raw experimental data and thermodynamic data such as entropy can be calculated as well as kinetic parameters such as activation energy, reaction order, and rate constant. The program accepts the reaction degree as ‘1’ for the Broido kinetic method and the flow diagram is given in Figure 1c.

#### 2.1.3. Application of PC program to NaNO3

In this study, the kinetics of the pyrolysis reaction of NaNO_3_ was examined as a reference and the program written in the ‘MATLAB 5.2’ environment was tested. TG/DTG/DTA analyses were performed in a differential thermal analyzer with analytical grade NaNO_3_ and α-Al_2_O_3_ reference material obtained from Merck.

Seventeen milligrams of sample was used for the measurement made at 10 K min^−1^ N_2_ gas flow rate and dynamic nitrogen atmosphere using a Pt reaction vessel. The TG/DTG/DTA diagrams of the NaNO_3_ compound are given in Figure 1d and the graph of the pyrolysis reaction according to the Broido method is given in Figure 1e. It was determined that the energy value calculated for NaNO_3_ (51.2 kJ/mol) was consistent with that in the literature (45.3 kJ/mol).

## 3. Results

### 3.1. Implementation of the software program for the thermal analysis data of N,N-di-n-butyl-N’-(4-chloro-benzoyl)thiourea ligand (HL) according to the Broido method

TG/DTA/DTG and GC/MS analyses were interpreted and it was determined that the ligand was decomposed in accordance with the reaction (3.1, 3.1a, 3.1b).


3.1

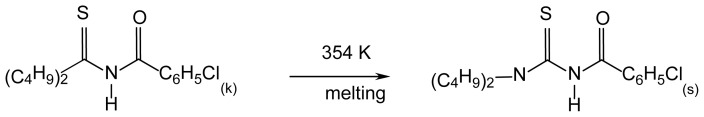



3.1a

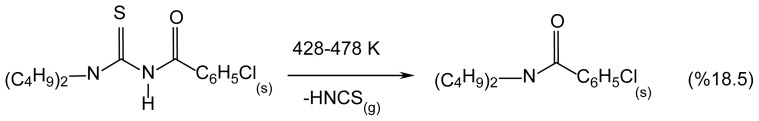



3.1b

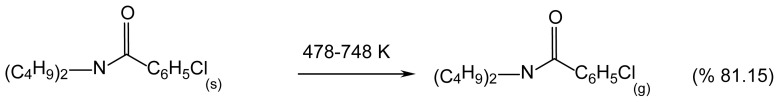


The decomposition kinetic data of the HL ligand calculated in the software program using the Broido method are given in Table 1 and the Broido graph is given in Figure 2a.

### 3.2. Implementation of software program according to Broido method for thermal decomposition data of the Bis(N,N-di-n-butyl-N’-(4-chloro-benzoyl)thioureato)nickel(II) [NiL2] complex

TG/DTA/DTG and GC/MS analyses were interpreted and it was determined that the ligand was degraded in accordance with the reaction (3.2.a, 3.2.b).


3.2.a $$\text{Ni}{\left({\text{ClC}}_{6}{\text{H}}_{4}\text{CONCSN}{\left({\text{C}}_{4}{\text{H}}_{9}\right)}_{2}\right]}_{2}\overset{490-577\;\text{K}}{\underset{-2({\text{ClC}}_{6}{\text{H}}_{4}\text{CONCSN}{\left({\text{C}}_{4}{\text{H}}_{9}\right)}_{2})}{\to }}\;\text{Ni}{\left(\text{SCN}\right)}_{2}$$

3.2.b $$\text{Ni}{\left(\text{SCN}\right)}_{2}\overset{570-581\;\text{K}}{\underset{-(\text{SCN}+\text{CN})}{\to }}\;\text{NiS}$$

The decomposition kinetic data of the [NiL_2_] complex, calculated in the software program using the Broido method, are given in Table 2 and the Broido graph is given in Figure 2b.

## 4. Discussion

The calculation of thermodynamic parameters using the Broido method is frequently encountered in the literature. For example, in the studies by Meena and Sharma, copper(II) soap complex was synthesized and kinetic parameters were calculated by using this equation. The present study revealed that the values of energy of activation for all the equations applied follow the order: Step III > Step II > Step I [18]. Hai et al. synthesized N-acryloyl-N-phenylthiourea. Thermal degradation kinetic parameters are determined for polymer samples From TGA curves using Broido’s, Coats–Redfern and Horowitz-Mitzger methods which provide overall kinetic data. The present study revealed that the values of energy of activation for all the equations applied follow the order: Step II > Step I [19]. A series of functionalized phenolformaldehyde polymer resins have been synthesized by the reactionof 2,4-dihydroxyacetophenone-formaldehyde resin with theamines, such as ethanolamine, aminophenol, ethylenediamine, and propylenediamine in dichloromethane. The calculated values for the activation energy of decomposition are 13.86, 9.98, 14.55, and 9.98 kJ/mol for DAPF-ea, DAPF-ap, DAPF-en, and DAPF-pn, respectively [20].

In this study, the decomposition mechanism and kinetics of the previously synthesized and characterized *N,N*-di-n-butyl-*N’*-(4-chloro-benzoyl)thiourea (HL) ligand and the bis(*N,N*-di-n-butyl-*N*’-(4-chloro-benzoyl)thioureato)nickel(II) [NiL_2_] complex were investigated using TG/DTG/DTA and GC-MS combined systems.

TG/DTG/DTA analyses were performed in a differential thermal analyzer. Analytical grade NaNO_3_ and α-Al_2_O_3_ were used as reference materials. Seventeen milligrams of sample was used and the measurements were made under 10 mL/min N_2_ gas flow rate using a Pt reaction pan. It is seen that the calculated energy value (51.2 kJ/mol) for NaNO_3_ is compatible with that in the literature (45.3 kJ/mol). It was determined that there is an absolute error of 1.1% between the values in the literature and the calculated values of the NaNO_3_ compound. This error is thought to be caused by the calibration of the device and the purity level of the substance. Thus, the accuracy of the kinetic parameters calculated in the program written in MATLAB 5.2 environment according to the Broido method of the decomposition reactions with the NaNO_3_ compound was tested.

Calculated kinetic parameters (E^*^, S, A) of the HL ligand and its Ni(II) complex obtained with the prepared PC software are given in Tables 1 and 2. The calculation method of the prepared PC software can be used with other integral equations besides the Broido equation with some modifications to the software. In addition, the results obtained for the compounds examined in this study can be compared with the results obtained with other methods.

## Figures and Tables

**Figure 1a:**
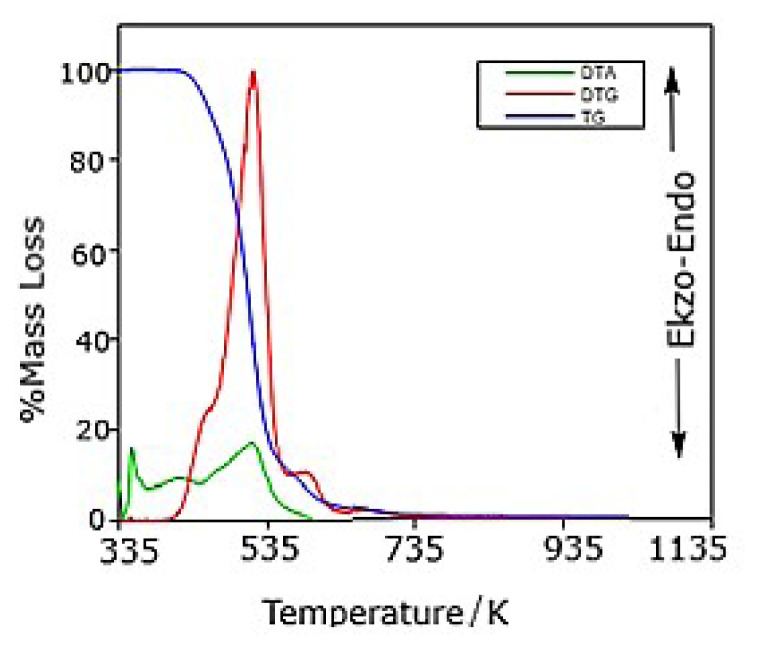
DTA/TG/DTG diagrams of HL.

**Figure 1b:**
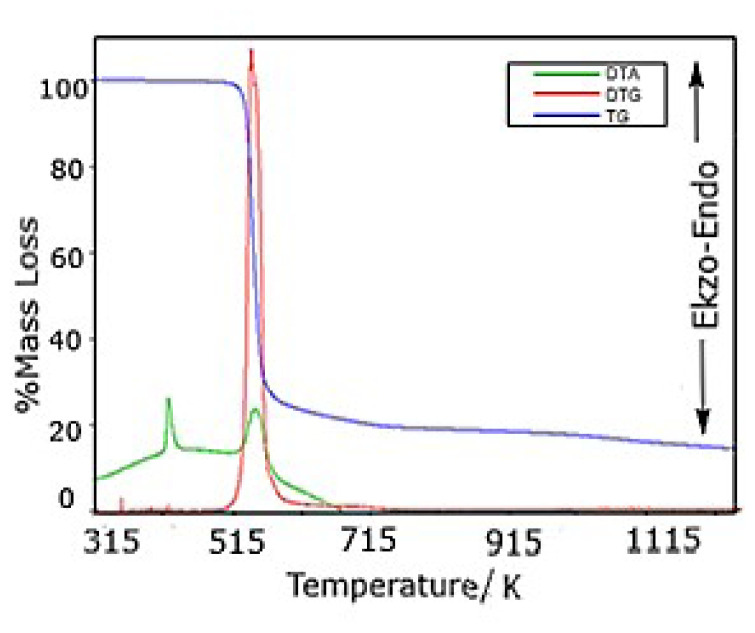
DTA/TG/DTG diagrams of [NiL_2_] complex.

**Figure 1c:**
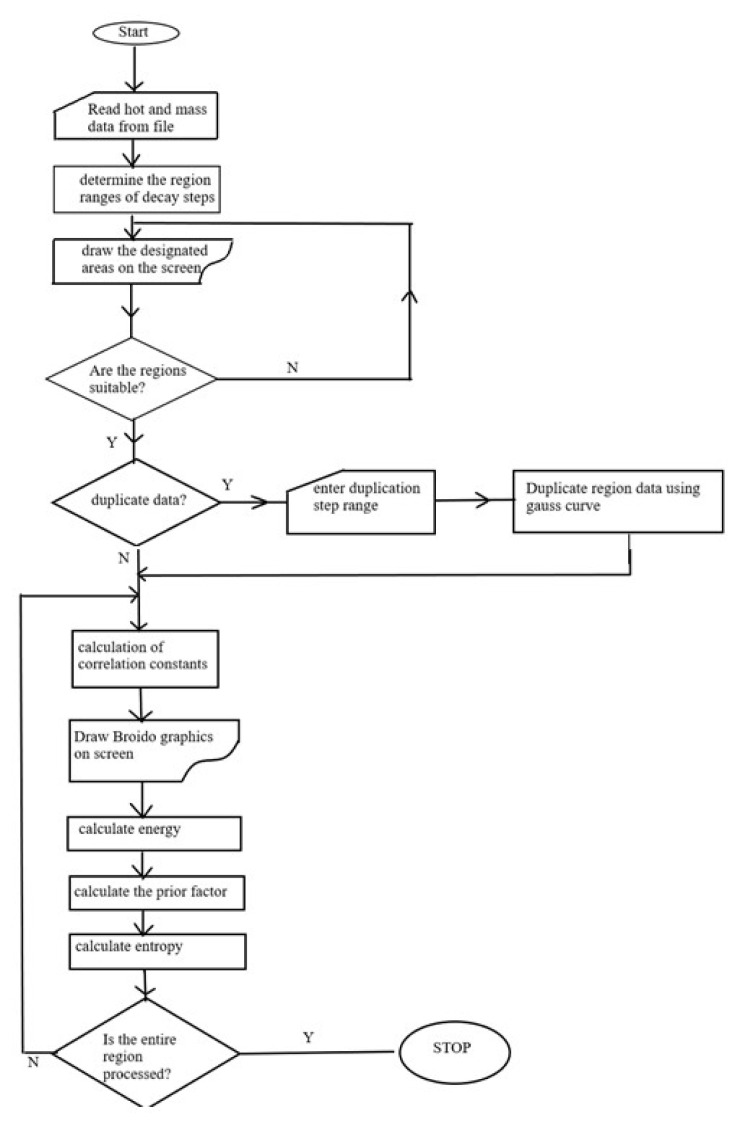
Flow diagram of the program written based on the broido kinetic method.

**Figure 1d:**
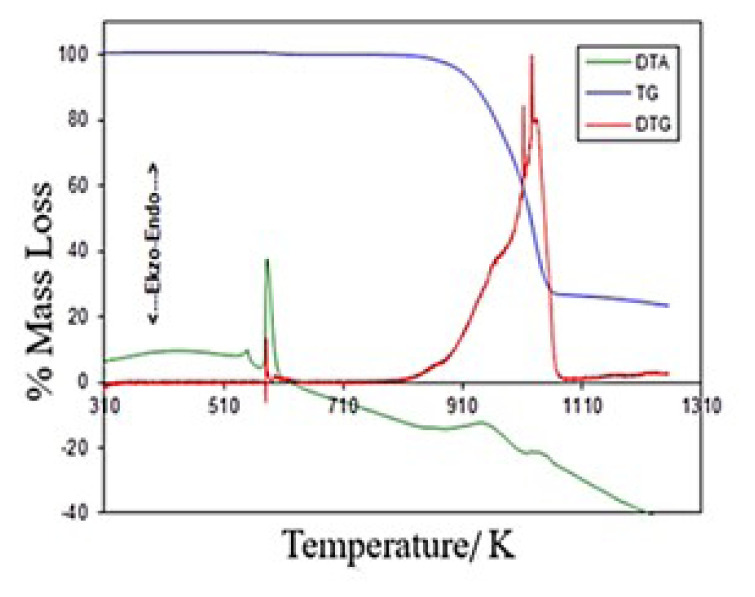
TG/DTG/DTA diagram of NaNO_3_.

**Figure 1e:**
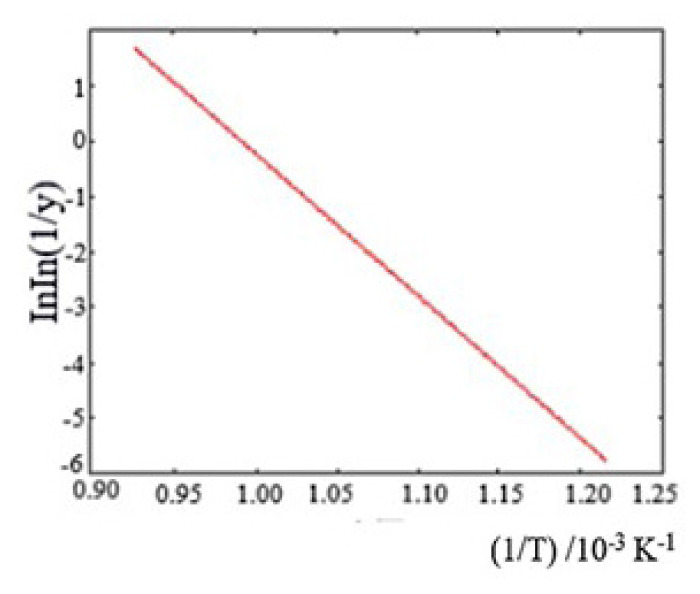
Broido graph of NaNO_3._

**Figure 2a:**
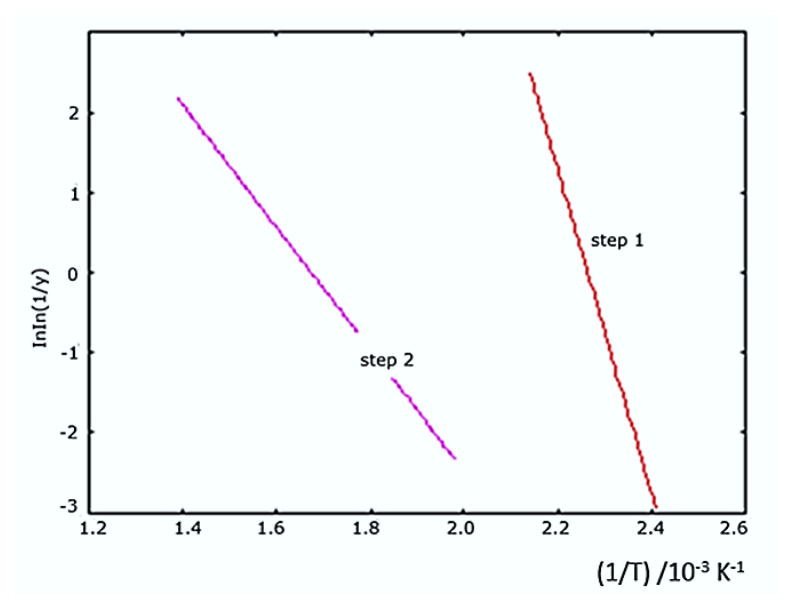
Broido plot for degradation steps of HL ligand.

**Figure 2b:**
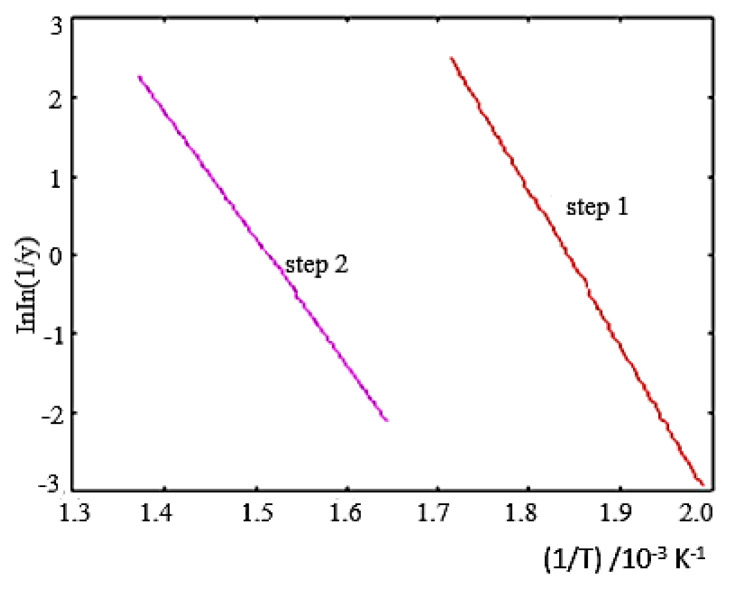
Broido plot for the degradation steps of the [NiL_2_] complex.

**Table 1:** Kinetic parameters for the decomposition steps of the HL ligand.

Compound	Decomposition step	Reaction degree	Parameters ^a,b^	Broido method
HL	I	1	E^*^	40.34
			A	0.22
			S	−211.8
			r	−0.92
	II		E^*^	15.19
			A	1.12
			S	−154.3
			r	−0.95

E^*^: kJ/mol, S: Jmol/K, A: 1/s

**Table 2:** Kinetic parameters for the decomposition steps of the [NiL_2_] complex.

Compound	Decomposition step	Reaction degree	Parameters ^a,b^	Broido method
[NiL_2_ ]	I	1	E^*^	39.26
			A	0.3
			S	−193.34
			r	−0.93
	II		E^*^	32
			A	0.21
			S	−171.54
			r	−0.93

E^*^: kJ/mol, S: Jmol/K, A: 1/s

## References

[1] Mackenzie RC Nomenclature in thermal analysis, part IV Thermochimica Acta 1979 28 1 1 6

[2] Pope MI Differential Thermal Analysis–A Guide to Technique and its Applications 1977

[3] Achar BN Brindley GW Sharp JH Kinetics and mechanism of dehydroxylation processes. III. Applications and limitations of dynamic methods Proc Int Clay Conf Jerusalem 1966 s67 73

[4] Sharp JH Wentworth SA Kinetic analysis of thermogravimetric data Analytical Chemistry 1969 41 14 2060 2

[5] Carroll B Manche EP Kinetic analysis of chemical reactions for non-isothermal procedures Thermochim Acta 1972 3 6 449 59

[6] Freeman ES Carroll B The application of thermoanalytical techniques to reaction kinetics: the thermogravimetric evaluation of the kinetics of the decomposition of calcium oxalate monohydrate Journal of Physical Chemistry 1958 62 4 394 7

[7] Brown ME Introduction to thermal analysis: techniques and applications C1 Springer Science & Business Media 2001

[8] Kayhan E Development of Software Programs Suitable for Calculation of Kinetic Parameters from Solid Phase Reaction Data, Application of Thiourea Derivatives to be Synthesized and some of their Complexes [PhD Thesis] Master Thesis Mersin University, Graduate School of Natural and Applied Sciences Mersin, Turkey 2003

[9] Arslan H FLÖRKE U Külcü N Kayhan E Synthesis, characterization, crystal structure and thermal behavior of N’-(4-chlorobenzoyl)-N, N-di-n-butylthiourea and its nickel complex Turkish Journal of Chemistry 2006 30 4 429 40

[10] Pekacar AI Özcan E Synthesis and complex formation of new unsymmetrical vic-dioximes Synthesis and Reactivity in Inorganic and Metal-Organic Chemistry 1995 25 6 0859 68

[11] Jones MEB Thornton DA Webb RF Metal-containing polymers. I. The preparation of bis (1, 2-dioximes) Macromolecular Chemistry and Physics 1961 49 1 62 8

[12] Grundmann C Mini V Dean JM Frommeld H-D Dicyan-di-N-oxyd Annual Reports on the Progress of Chemistry 1965 687 1 191 214

[13] Lrez G Bekaroglu Ö The synthesis and complex formation of swe new substituted amino and diaminoglyoximes Synthesis and Reactivity in Inorganic and Metal-Organic Chemistry 1983 13 6 781 97

[14] Arslan H Özpozan N Tarkan N Kinetic analysis of thermogravimetric data of p-toluidino-p-chlorophenylglyoxime and of some complexes Thermochimica Acta 2002 383 1–2 69 77

[15] Arslan H Külcü N Pekacar Aİ Thermal decomposition kinetics of anilino-p-chlorophenylglyoxime complexes of cobalt (II), nickel (II) and copper (II) Turkish Journal of Chemistry 2003 27 1 55 64

[16] Burakevich JV Lore AM Volpp GP Phenylglyoxime. Separation, characterization, and structure of three isomers The Journal of Organic Chemistry 1971 36 1 1 4

[17] Broido A A simple, sensitive graphical method of treating thermogravimetric analysis data Journal of Polymer Science Part B: Polymer Physics 1969 7 10 1761 73

[18] Meena A Sharma R Degradation Kinetics of Copper (ii) Sesame thiourea complex Emerging Sustainable Technologies and Innovations for Safe Water and Health.14 th Biyani International Conference (BICON-19) 978-93-83462-95-7

[19] Hai FA Whhab HA Ahmed GI Synthesis, characterization and biological activity of thiourea contaınıng polymers Al-Azhar Bulletin of Science 2013 24 1 June 1 15

[20] Reddy AR Reddy KH Synthesis of functionalized phenolformaldehyde polymer resins by the reaction of 2,4-Dihydroxyacetophenoneformaldehyde resin with various amines and their metal ion uptake properties Journal of Applied Polymer Science 2004 92 1501 1509

